# Retraction: Impaired Insulin Signaling Affects Renal Organic Anion Transporter 3 (Oat3) Function in Streptozotocin-Induced Diabetic Rats

**DOI:** 10.1371/journal.pone.0202898

**Published:** 2018-08-21

**Authors:** The PLOS ONE Editors

Concerns were raised about similarities between the following figure panels in this *PLOS ONE* article [1] and some of the same authors’ prior publication in the *American Journal of the Medical Sciences* [2]:

- Figure 2A [1], β-actin blot lanes 2, 3, and Figure 4C [2], β-actin blot lanes 1, 2.
- Figure 2A [1], Oat3 blot lanes 1, 2 and Figure 6C [2], phospho-PKCα blot lanes 2, 3.

Furthermore, the Figure 2C β-actin blot lanes 3, 2 in the *PLOS ONE* article appear to overlap with the Figure 2A β-actin blot lanes 2, 3 following 180-degree rotation. We also noted potential image irregularities in Figure 2A [1], Na^+^-K^+^-ATPase blot lane 1.

The corresponding author noted that the image panels for which concerns were raised present images from independent experiments; the original underlying blots are not available. Given specific image details in the above figures and the unavailability of the supporting primary data, the *PLOS ONE* Editors remain concerned about the noted similarities.

In addition, the first three subsections of the Results in [1] show significant overlap with experiments and results previously reported in [2]. While the *American Journal of the Medical Sciences* article was discussed in the Introduction and Discussion of the *PLOS ONE* article and cited in the Methods, this overlap was not acknowledged in the Results.

In light of the image concerns, data unavailability, and partial redundancy with previously published work, the *PLOS ONE* Editors retract this article.

AL agreed with the retraction. PA, AP, CS, LC, VC did not respond.
